# Optimization of drying conditions for microwave-vacuum drying of tomato snack bars using response surface methodology

**DOI:** 10.1016/j.fochx.2026.104365

**Published:** 2026-08-28

**Authors:** Muhammed Rasim Gul, Nalan Yazicioglu, Gulum Sumnu, Mecit Halil Oztop

**Affiliations:** a Department of Food Engineering, Middle East Technical University, Ankara 06800, Türkiye; b Department of Nutrition and Dietetics, Gulhane Faculty of Health Sciences, University of Health Sciences, Ankara 06018, Türkiye; c BIOMATEN, METU Research Center, Middle East Technical University, 06800 Ankara, Türkiye

**Keywords:** Microwave vacuum drying, Response surface methodology, Tomato snack bars, Physicochemical properties, Box-Behnken design

## Abstract

The study focused on optimizing microwave-vacuum drying (MVD) for tomato snack bars using response surface methodology (RSM). Key variables included drying time, microwave power, and absolute chamber pressure, with a Box-Behnken design testing power levels (1.0, 1.2, 1.4 kW), pressures (0.2, 0.35, 0.5 bar), and drying times (8, 10, 12 min). Targets for water activity (0.50), moisture content (15%), and hardness (45 N) were set based on comparable studies in snack bar literature. The goal was to maximize lycopene and redness, indicated by the *a*^⁎^/*b*^⁎^ value, whereas energy demand was calculated as a deterministic objective. The optimal parameters were identified as 1.28 kW power, 0.46 bar pressure, and 9.2 min drying time, achieving a desirability of 0.83. At the selected condition, the experimental values were water activity (0.494), moisture content (15.15%), lycopene (216.5 mg/kg), redness (*a*^⁎^/*b*^⁎^) of 0.806, hardness (54.35 N), and the corresponding theoretical energy estimate was 1424 kJ.

## Highlights

• MVD parameters were optimized for the production of tomato-based snack bars.
• MVD achieved target moisture and water activity under the selected conditions.
• The selected condition yielded 216.5 mg/kg dry matter lycopene and *a*^⁎^/*b*^⁎^ 0.806.
• Energy was treated as a deterministic rather than an empirical RSM objective.

## 1 Introduction

The primary objective of tomato snack bar production is to reintroduce the Mediterranean diet to consumers and to create healthy, high-nutritional-value snacks. Microwave vacuum drying (MVD) technology was used for production.

Vacuum drying can convert fresh fruits like carrots and bananas into shelf-stable, nutrient-rich snack bars (Ignaczak et al., 2023). This segment is rapidly growing as consumers seek healthy, functional snacks with added benefits such as fiber, protein, and antioxidants (Santiago-Ramos et al., 2022). Manufacturers are increasingly incorporating fruits and vegetables to meet this demand (Ahaotu et al., 2022). Different drying methods, including hot-air drying, freeze-drying, and infrared drying, are utilized to lower moisture content and achieve low water activity (∼0.6), ensuring shelf stability without compromising quality (Gul et al., 2024).

Tomato-based snacks offer both flavor and health benefits due to their high content of bioactive compounds like lycopene, an antioxidant linked to reduced risks of various diseases (Santiago-Ramos et al., 2022). While fresh tomatoes are perishable, drying methods such as vacuum-assisted microwave drying effectively preserve nutrients, improving flavor, color, and texture compared to traditional hot-air drying (Gul et al., 2024). Optimizing parameters such as microwave power and vacuum level is crucial for achieving optimal results, and Response Surface Methodology (RSM) is a useful tool for determining the optimal conditions (Alvi et al., 2023). This study aims to optimize drying conditions for vacuum-assisted microwave-drying of tomato snack bars, focusing on quality and efficiency.

Microwave–vacuum drying (MVD) combines vacuum and microwave drying techniques. It operates under low pressure, allowing high-energy water molecules to evaporate while preventing oxidation, thus preserving the color, texture, and flavor of dried products (Punathil & Basak, 2016). MVD achieves water removal at lower temperatures and shorter processing times, reducing undesirable changes in sensory qualities and nutritional value (Wardhani et al., 2022). Additionally, it enhances microbial reduction through its low-temperature, quick processing.

Microwave vacuum drying (MVD) offers several benefits over traditional hot-air drying, such as faster drying times and reduced overheating in low-moisture areas (Wardhani et al., 2022). However, accurate moisture diffusion models are essential for broader industrial applications (Dak & Pareek, 2014). MVD can heat food unevenly, similar to other microwave processes, but it effectively preserves nutrients. For example, in apple slices, MVD achieved up to a 51% greater apparent volume and nearly doubled the phenolic content at 4.5 W/g compared to air-drying methods (Bulantekin & Kuşçu, 2024). Additionally, advanced dehydration techniques like MVD enhance energy efficiency and nutrient retention (Ates et al., 2025; Radojčin et al., 2021). Although energy-efficient since heat is generated within the food (Suárez et al., 2000), the method faces challenges, including high installation costs and potential non-uniform heating in thicker materials, which may affect product quality (Tzempelikos et al., 2014).

The MVD technique has been effectively utilized to dry various fruits, including sour cherry pomace and pitaya peel (Chew & King, 2019; Wojdyło et al., 2014). Vacuum drying mitigates overheating by evaporating moisture at lower temperatures, preserving bioactive compounds (Kayisoglu & Ertekin, 2011). Optimizing drying conditions is vital for higher lycopene content (Kong et al., 2010). Another study employed a Box-Behnken design (BBD) to evaluate the drying efficiency of MVD on kiwifruit (Tian et al., 2015). Although Gul et al. (2024) have demonstrated the feasibility of MVD for tomato snack bars, the interactions between drying parameters and product quality remain unexplored. This research fills this gap by systematically optimizing the MVD process, focusing on how vacuum application and microwave power preserve heat-sensitive lycopene and create a stable, high-protein structure. It also defines the quality-efficiency boundary and determines the processing timeframe for large-scale production of shelf-stable tomato snack bars.

Therefore, this study aimed to optimize microwave-vacuum drying conditions for tomato snack bars to maximize lycopene and redness, and meet targets for moisture, water activity, and hardness. The corresponding theoretical energy estimate was also calculated. MVD settings were optimized using response surface methodology in MINITAB and Origin by adjusting microwave power, absolute chamber pressure, and drying time.

## 2 Material & methods

### 2.1 Preparation of the snack bar

**Fig. 1:**
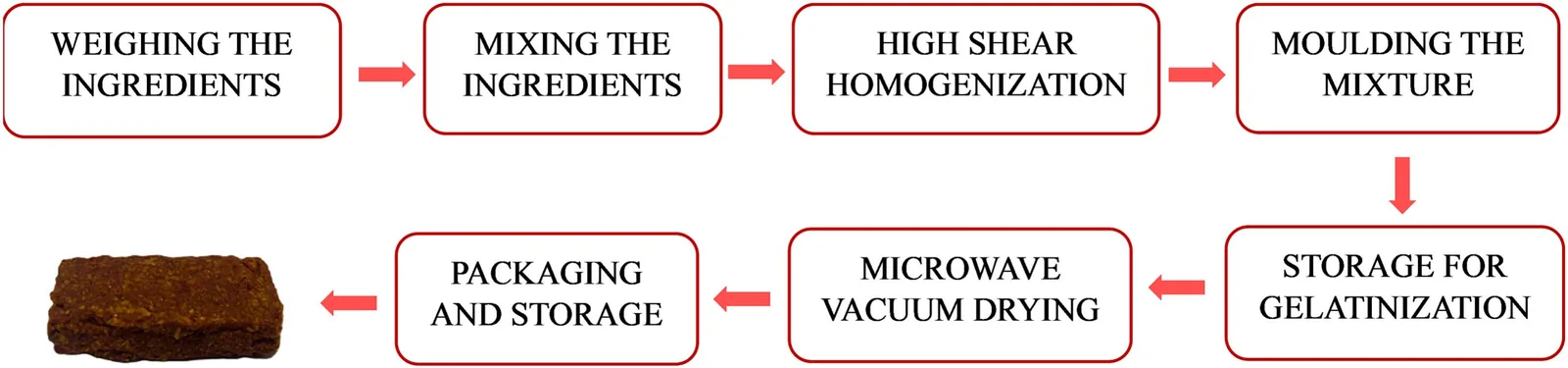
Flow chart of the production of a tomato snack bar.

The flowchart of the production process is shown in Fig. 1. The composition of the tomato snack bars was chosen based on a previous study (Gul et al., 2024). The ingredients used were 100 g of tomato juice, 5 g of tomato powder, 5 g of tomato peel powder, 1 g of low-methoxylated pectin (LMP, 27%), 10 g of pea protein, 2 g of salt, 2 g of olive powder, and 1 g of each seasoning, which are thyme, rosemary, and ground red pepper.

To prepare the product, all ingredients were first weighed using a precise scale. Tomato juice from fresh tomatoes (Kraft Heinz Food, Balıkesir, Turkey) was mixed with LMP (Cargill, Balıkesir, Turkey) using a high-shear homogenizer (Ultraturrax T25, IKA Werke, Staufen, Germany) for about 2.5 min. Afterward, pea protein (Vegrano, Başakşehir, Istanbul, Turkey) and salt were added and homogenized until uniform. Seasonings from the local market and freeze-dried olive powder from homogenized green olives were then incorporated, mixed by hand, and poured into bar molds. The bars cooled overnight at 4 °C to form a gel, then dried with a microwave vacuum drying system.

### 2.2 Experimental design for the drying of the snack bar

#### 2.2.1 Drying of the snack bar

A microwave-vacuum oven (2 kW, 2450 MHz, Plazmatek, Isparta, Turkey) equipped with an oil-sealed rotary vacuum pump (2.4 kW motor power, Model VDN601, ULVAC, China) was used to dry the bars. Microwave power (1.0, 1.2, 1.4 kW), absolute chamber pressure (0.2, 0.35, 0.5 bar), and drying time (8, 10, 12 min) were investigated using a Box-Behnken response surface design. Lower abs. (absolute) chamber pressure denotes a stronger vacuum in the plots.

#### 2.2.2 Optimization procedure

The optimization of MWV drying for tomato snack bars was carried out using Response Surface Methodology (RSM). The experimental design used was the Box- Behnken design, prepared with MINITAB 19 software (Minitab Inc., State College, PA, USA). As shown in Table 2, the Box- Behnken design included three levels and three factors, with two replicates, resulting in 30 experimental runs. Response surface plots were generated with Origin 2018 software (OriginLab Corporation, Northampton, MA, USA). The three independent variables considered were microwave power (A: 1.0, 1.2, 1.4 kW), absolute chamber pressure (B: 0.2, 0.35, 0.5 bar), and drying time (C: 8, 10, 12 min). The dependent variables measured were water activity, moisture content, lycopene content, color *a*^⁎^/*b*^⁎^ ratio, and texture hardness. A theoretical energy estimate was calculated from equipment-rated power and process time, as it was deterministic. The experiment aimed to achieve target values for water activity (0.50), moisture content (15%), and texture hardness (45 N), while maximizing lycopene content and redness (color *a*^⁎^/*b*^⁎^). Recent studies have helped develop functional tomato snack bars (Ayhan et al., 2025; Tomassi et al., 2025). Gul et al. (2024) stated that a water activity of 0.6 or lower is necessary to inhibit microbial growth and extend shelf life. Additionally, acceptable residual moisture (14–20%) was achieved at a water activity of 0.6. Maintaining lycopene and color is essential for health benefits and visual appeal (Santiago-Ramos et al., 2022). Texture, especially hardness, affects ease of eating for infants and the elderly. Gul et al. (2024) indicated that optimal hardness was around 45 N. Ayhan et al. (2025) emphasized that lower energy consumption improves life-cycle assessment scores. Energy calculation was also used as a deterministic objective function constraint in optimization. The study aimed to optimize drying parameters to produce microbially stable, health-promoting, and attractive tomato snack bars using RSM. Quadratic models fitted response equations (Table 3). Validation of the model was done by performing an experiment at the optimum factor levels predicted by the desirability function (MW Power: 1.28 kW, absolute chamber pressure: 0.46 bar, drying time: 9.2 min). The experimental values obtained for the five responses were compared with their corresponding predicted values to evaluate the predictive power of the established quadratic models. The Root Mean Square Error (RMSE) of the five responses, excluding energy, was low (4.83), indicating close overall agreement between the experimental and predicted data sets (Table 4).

### 2.3 Selection of variables

Selecting independent parameters is crucial for determining the physicochemical properties of snack bars. Factors include microwave power, absolute chamber pressure, drying time, and initial moisture content (Han et al., 2010). This study used a tomato snack bar to identify optimization ranges. Drying time, microwave power, and absolute chamber pressure levels were based on prior research. The snack was dried under extreme conditions: 2 kW microwave power, 20 min of drying, and 0.1 bar absolute chamber pressure, with all other conditions held constant. Range selection aimed for a water activity of 0.5. Another study maximized lycopene content in dried products (Abano et al., 2013).

The target values for optimization were based on literature and balancing key factors from snack bar studies. A water activity (*a*_w_) of 0.50 was set, following the study on tomato, papaya, and guava snack bars by Shivani et al. (2026), as values below 0.60 inhibit spoilage bacteria, yeasts, and molds (Tapia et al., 2008). The moisture content target of 15% (db) was chosen for chemical stability and desirable chewy texture (Labuza & Altunakar, 2020). A hardness of 45 N was selected based on a study on amaranth-based honey bars (Kigozi et al., 2024), emphasizing the need for structural integrity and ease of eating. The initial non-optimized formulation served as the control, with Response Surface Methodology used to optimize the product for shelf stability.

### 2.4 Analyses of tomato snack bars

#### 2.4.1 Moisture content and water activity (*a*_w_)

The moisture content (MC) of the samples was determined gravimetrically by drying the samples at 105 °C in an oven. The *a*_w_ was determined by using Aqua Lab 4TE Dew Point & Water Activity Meter (Decagon Devices Inc., Pullman, Wash., U.S.A.) at 25 °C.

#### 2.4.2 Color

CIE *L*^⁎^*a*^⁎^*b*^⁎^ values were measured with a portable spectrocolorimeter (Serlab SL400, Istanbul, Turkey) at three points on one sample, and the averages were reported. Pék et al. (2010) noted that the *a*^⁎^/*b*^⁎^ ratio is the most common parameter to assess color during tomato ripening. The *a*^⁎^ value indicates green/red, *b*^⁎^ indicates blue/yellow, and the ratio reflects redness relative to yellowness. Thus, the *a*^⁎^/*b*^⁎^ ratio was evaluated.

#### 2.4.3 Texture analysis

Texture Profile Analysis (TPA) was conducted using a texture analyzer (CT3 Brookfield, Middleboro, USA) with at least three replicates, following the reference method of Gul et al. (2024). Hardness (N) data were obtained from the TPA diagram.

#### 2.4.4 Lycopene content

Lycopene content was measured using the method described by Gul et al. (2024) with three replicates. UV–Vis spectrophotometry (Optizen POP UV–Visible, Mecasys, Daejeon, Korea) was employed.

#### 2.4.5 Energy consumption calculation

The theoretical estimation of total energy expenditure (*E*_*total*_, kJ) for the MVD process was calculated by integrating the power consumption of the microwave generator (*P*_*mw*_) and vacuum pump (*P*_*vac*_) over time (*t*, s). To account for the varying load on the vacuum system, the pump power was modeled as a function of the absolute chamber pressure in Eq. (1) below:

(1) $$\;P_{\mathrm{vac}}=P_{\max}*\left(1-P_{\mathrm{abs}}\right)$$

where *P*_*max*_ is the maximum rated power of the vacuum pump (2.4 kW), and *P*_*abs*_ is the absolute chamber pressure expressed in bar. The total energy was then calculated as in Eq. (2):

(2) $$\;E_{\mathrm{total}}=\left(P_{\mathrm{mw}}+P_{\mathrm{vac}}\right)*t$$

These are calculation assumptions rather than direct power-meter measurements. *E*_*total*_ was consequently used only as a deterministic minimization objective.

## 3 Results & discussion

### 3.1 Selection of independent variables

Water activity correlates with free water evaporation in dried products (Gul et al., 2024). Very low microwave power or brief drying times failed to ensure microbial stability in snack bars due to high water activity levels (>0.7). Increasing power and time decreased water activity but made bars too tough for the elderly. Vacuum drying can shorten duration with higher vacuum levels. The pressure factor was recorded as absolute chamber pressure; thus, 0.2 bar represents stronger vacuum than 0.5 bar. Therefore, the selected design region was intended to balance evaporation, product quality, and process duration. All parameters must be optimized for target water activity and texture.

Increasing lycopene in dried tomato products is also desirable. Heat processing releases lycopene from the cell matrix, and the content rises with processing temperatures up to 80 °C (Gul et al., 2024). Extending drying time and increasing temperature can boost lycopene levels (Mendelová et al., 2013), but higher temperatures may cause degradation due to faster lycopene loss with excessive microwave power or drying time. Abano et al. (2013) stated that higher vacuum levels increase lycopene content. Several studies have also shown that porosity and sensory qualities can be improved by lowering drying temperature and promoting puffing (Calín-Sánchez et al., 2020; Cong et al., 2023; Gong et al., 2025; Nguyen & Marzec, 2024; Wardhani et al., 2022). Measured lycopene can reflect competing effects during tomato processing. Matrix disruption and moisture concentration can increase the analytical extractability, while thermal exposure, oxygen, and localized microwave heating can promote isomerization or degradation (Abano et al., 2013; Colle et al., 2010; Heredia et al., 2010). Reduced pressure may shorten exposure by lowering the boiling temperature, but it does not guarantee pigment retention under every microwave power-time combination. Therefore, the optimization used measured lycopene content together with color, water removal, and texture. No claim of improved lycopene bioavailability was made, as bioaccessibility was not measured. Conversely, high temperatures and long durations can create indigestible or tough textures via protein aggregation and Maillard browning (Zhou et al., 2013). These findings indicate that extremely high temperatures are not always beneficial for lycopene content or product quality. Previous studies have also demonstrated better lycopene content in snack bars processed at 1–1.5 kW power, under a 0.3 bar vacuum, and for about 10 min of drying. However, optimizing drying still considers color, texture, and moisture.

### 3.2 Fitting the model for the experimental data

A Box-Behnken design examined how microwave power, absolute chamber pressure, and drying time affect the experimentally measured responses to optimize tomato snack bar drying. The Box–Behnken design used coded levels (−1, 0, +1), with actual values in Table 1. Regression equations in Table 3 are in actual units (kW, bar, min). Response surface plots show actual variable values for clarity. Data and responses are in Table 2. The quadratic model analyzed interactions, and ANOVA was conducted at 95% confidence. A good model fit requires a significant *p*-value indicating the fitted terms explain response variation. An insignificant lack-of-fit is also required; otherwise, a significant lack-of-fit *p*-value suggests the second-order polynomial doesn't adequately represent the response over the full design region. The R^2^, adjusted R^2^, predicted R^2^, and sum of squares evaluate model adequacy and suitability.

**Table 1:** Independent variables and their coded/actual levels for the Box-Behnken design.

Factors	Coded: −1	Coded: 0	Coded: +1
A: Microwave Power (kW)	1.0	1.2	1.4
B: Abs. Chamber Pressure (bar)	0.2	0.35	0.5
C: Drying Time (min)	8	10	12

*Note: B is absolute chamber pressure; a lower B value corresponds to a stronger vacuum*.

**Table 2:** Factor settings, measured responses, and calculated energy objective for the Box–Behnken design.

	Factors			Measured responses					Calculated objective
Run	A: MW power (kW)	B: Absolute pressure (bar)	C: Drying time (min)	*a*_w_	Moisture (% db)	*a*^⁎^/*b*^⁎^	Lycopene (mg/kg dry)	Hardness (N)	Energy estimate (kJ)
1	1	0.2	10	0.436	13.40	0.777	182.72	28.40	1716
2	1	0.2	10	0.426	12.78	0.781	165.98	24.84	1716
3	1	0.5	10	0.674	24.74	0.803	209.90	14.71	1320

4	1	0.5	10	0.632	20.28	0.814	214.62	15.11	1320
5	1	0.35	8	0.808	30.46	0.837	208.62	14.71	1214
6	1	0.35	8	0.740	32.56	0.827	209.04	14.92	1214

7	1	0.35	12	0.385	11.12	0.798	170.56	78.01	1822
8	1	0.35	12	0.459	13.41	0.803	173.85	78.13	1822
9	1.2	0.2	8	0.535	16.09	0.815	170.13	20.21	1478

10	1.2	0.2	8	0.558	17.08	0.808	171.56	18.48	1478
11	1.2	0.5	8	0.707	22.27	0.804	244.24	27.02	1162
12	1.2	0.5	8	0.581	20.37	0.792	243.24	28.44	1162

13	1.2	0.2	12	0.322	9.04	0.790	159.25	90.75	2218
14	1.2	0.2	12	0.371	10.57	0.788	158.54	113.24	2218
15	1.2	0.5	12	0.383	11.06	0.766	178.28	100.76	1742

16	1.2	0.5	12	0.380	11.09	0.766	190.59	102.90	1742
17	1.2	0.35	10	0.512	15.32	0.834	156.10	76.41	1650
18	1.2	0.35	10	0.515	15.14	0.836	175.56	77.27	1650

19	1.2	0.35	10	0.478	13.70	0.829	176.42	73.18	1650
20	1.2	0.35	10	0.496	14.36	0.829	181.86	75.50	1650
21	1.2	0.35	10	0.505	13.44	0.839	165.55	75.51	1650

22	1.2	0.35	10	0.502	14.28	0.839	191.73	76.39	1650
23	1.4	0.2	10	0.331	9.20	0.841	142.08	83.12	1980
24	1.4	0.2	10	0.325	9.31	0.838	142.94	84.36	1980

25	1.4	0.5	10	0.351	10.93	0.812	210.05	34.54	1584
26	1.4	0.5	10	0.337	11.05	0.814	195.17	32.46	1584
27	1.4	0.35	8	0.436	13.20	0.786	183.72	29.60	1426

28	1.4	0.35	8	0.509	18.19	0.783	178.57	29.84	1426
29	1.4	0.35	12	0.355	11.48	0.874	120.48	58.57	2138
30	1.4	0.35	12	0.349	13.68	0.876	143.66	60.08	2138

*Note: Energy values are theoretical equipment-based estimates calculated from the factor settings; they are not experimental responses and were excluded from RSM model fitting*.

All five quadratic regressions were significant (model *p* < 0.001), but adequacy was response-dependent (Table 3). The *a*_w_, moisture content, and lycopene models had R^2^ values of 0.955, 0.95, and 0.93, respectively, with nonsignificant lack of fit (*p* = 0.441, 0.130, and 0.456). Therefore, these models were used for prediction within the studied factor ranges. In contrast, the *a*^⁎^/*b*^⁎^ and hardness models had significant lack of fit (both *p* < 0.001), although their R^2^ values were 0.810 and 0.857. Their surfaces were retained only to display empirical trends and were not interpreted as globally reliable predictive equations. Energy was not included in Table 3 as it was calculated deterministically rather than measured experimentally.

**Table 3:** Optimized response polynomial equations fitted in the quadratic model for the tomato snack bar.

Response	Quadratic equation (actual units)	R^2^	Adjusted R^2^	Model *p*-value	Lack-of-fit p-value
*a*_w_	*a*_w_ = 2.054–0.232*A* + 4.254*B* − 0.3252*C* − 0.460*A*^2^–1.948*B*^2^ + 0.00557*C*^2^–1.719*AB* + 0.1447*AC* − 0.0519*BC*	0.955	0.935	<0.001	0.441
Moisture (% db)	*MC* = 237.9–184.8*A* + 178.0*B* − 24.49*C* + 36.3*A*^2^–82.9*B*^2^ + 0.547*C*^2^–64.0*AB* + 10.08*AC* − 2.89*BC*	0.950	0.927	<0.001	0.130
*a*^*⁎*^/*b*^*⁎*^	*a*^*⁎*^/*b*^*⁎*^ = 1.070–0.769*A* + 1.477*B* − 0.0128*C* + 0.097*A*^2^–1.245*B*^2^–0.00377*C*^2^–0.467*AB* + 0.0758*AC* − 0.0073*BC*	0.810	0.725	<0.001	<0.001†
Lycopene (mg/kg dry)	*Lycopene* = 144 + 171*A* − 42*B* − 0.6*C* − 94.0*A*^2^ + 540*B*^2^ + 0.696*C*^2^ + 185*AB* − 7.78*AC* − 39.5*BC*	0.930	0.898	<0.001	0.456
Hardness (N)	*Hardness* = −1630 + 1965*A* + 704*B* + 61.3*C* − 666*A*^2^ − 417*B*^2^–0.90*C*^2^ − 321*AB* − 21.0 *AC* − 7.1*BC*	0.857	0.792	<0.001	<0.001†

*A*, *B*, and *C* are in actual units*. A = microwave power (kW); B = absolute chamber pressure (bar); C = drying time (min). Model p-values reported as 0.000 by the software are presented as <* *0.001. †Significant lack of fit; these equations are retained only as exploratory trend descriptions and not as globally adequate predictive models. Energy is excluded because it was calculated deterministically*.

### 3.3 Effect of independent variables on water activity

**Fig. 2:**
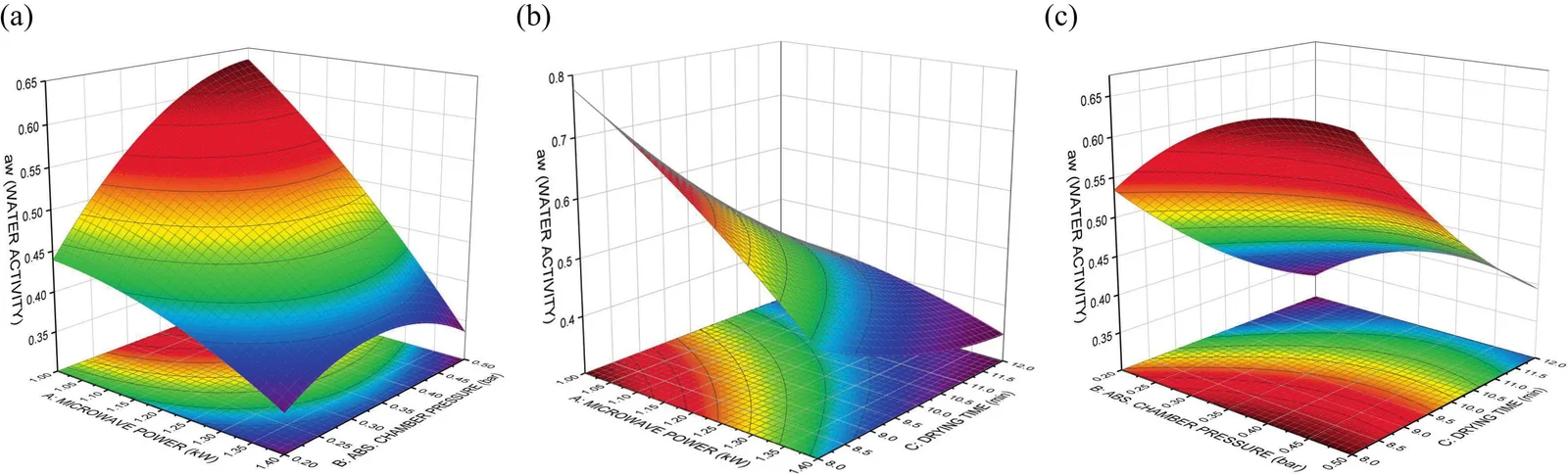
Response surface plots showing the effect of MWV drying parameters on water activity of tomato snack bar (a, b, and c).

The effect of the independent variables on the water activity (*a*_w_) of the snack bars is shown in Fig. 2. The quadratic model indicated that *a*_w_ depended on both the individual processing variables and their interactions. Absolute chamber pressure made a substantial contribution to the response, while its quadratic term and interaction with microwave power indicated a nonlinear and condition-dependent effect (Table 3).

Increasing absolute chamber pressure from 0.20 to 0.50 bar (i.e., weakening the vacuum) generally resulted in higher *a*_w_, especially at lower microwave power, which is consistent with slower moisture removal. The magnitude of the pressure effect decreased at higher microwave power, demonstrating interaction within the studied region. The observed curvature is interpreted as a process-specific interaction among heating, pressure, and time, not as a general rule that both pressure extremes inherently reduce *a*_w_.

Increasing microwave power generally reduced water activity, consistent with enhanced volumetric heating and faster drying. However, the magnitude of this effect depended on absolute chamber pressure as indicated by the (AB) interaction. The quadratic term of microwave power also has a minor curvature effect. Increasing microwave power and decreasing absolute chamber pressure (increasing vacuum) both reduce *a*_w_ (Fig. 2(a)). Longer drying times also generally decreased *a*_w_, while the small quadratic coefficient for drying time suggested limited curvature over the investigated range (Fig. 2(b) and Fig. 2(c)). The previous microwave-drying studies have similarly reported that greater microwave intensity, longer processing, and reduced pressure (stronger vacuum) can enhance moisture removal, although the magnitude of these effects depends on the food matrix and processing configuration (Horuz et al., 2017; Nisoa et al., 2021; Zeng et al., 2025).

Overall, microwave power, absolute chamber pressure, and drying time should be considered jointly to ensure adequate mass transfer and achieve the target water activity.

### 3.4 Effect of independent variables on moisture content

**Fig. 3:**
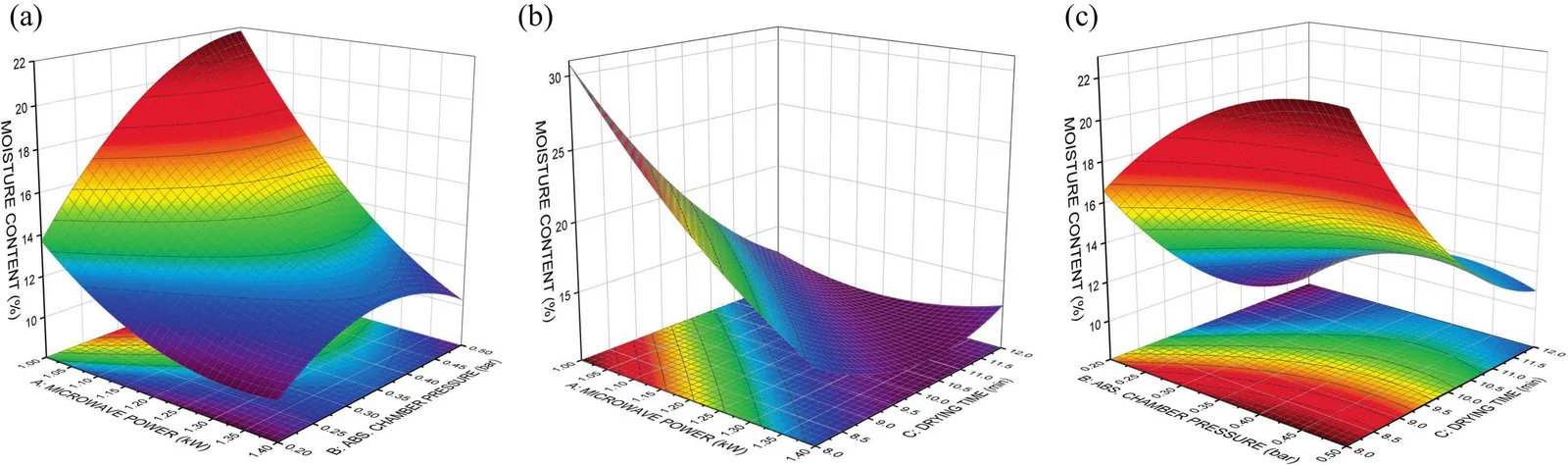
Response surface plots showing the effect of MWV drying parameters on moisture content (MC, %) of tomato snack bar (a, b, and c).

The 3D contour plots for microwave power (A), absolute chamber pressure (B), and drying time (C) show the moisture content of tomato snack bars in Fig. 3. Moisture content (MC) had a strong inverse relationship with microwave power and a weak inverse relationship with drying time; increasing these reduced MC. Higher microwave power caused rapid drying, making it the main factor, while longer drying times slightly decreased MC. Drying time had a modest effect, likely due to quick equilibrium, consistent with a study on mulberry microwave-vacuum drying (Cong et al., 2023). MC generally increased with higher absolute pressure (B), weaker vacuum, as higher pressure reduced evaporation and tended to retain more moisture at matched settings. The pressure effect was most evident at the lower microwave-power levels, indicating an interaction between microwave heating and the evaporation environment. A strong negative AB interaction (coefficient −64.0) caused MC to drop when A was high and B was low (Fig. 3(a)). Interactions like AC (10.1) and BC (−2.9) were minor. The highest observed MC was 32.56% dry basis at 1.0 kW, 0.35 bar, and 8 min, while the lowest was 9.04% dry basis at 1.2 kW, 0.20 bar, and 12 min. Values near the target (15.0%) were at the central parameters (1.2 kW, 0.35 bar, 10 min). All three parameters significantly affected moisture content. As the MC model had nonsignificant lack of fit (*p* = 0.130), the surface can be used for interpolation within the tested domain but not extrapolated beyond it.

### 3.5 Effect of independent variables on color *a*^⁎^/*b*^⁎^ value

**Fig. 4:**
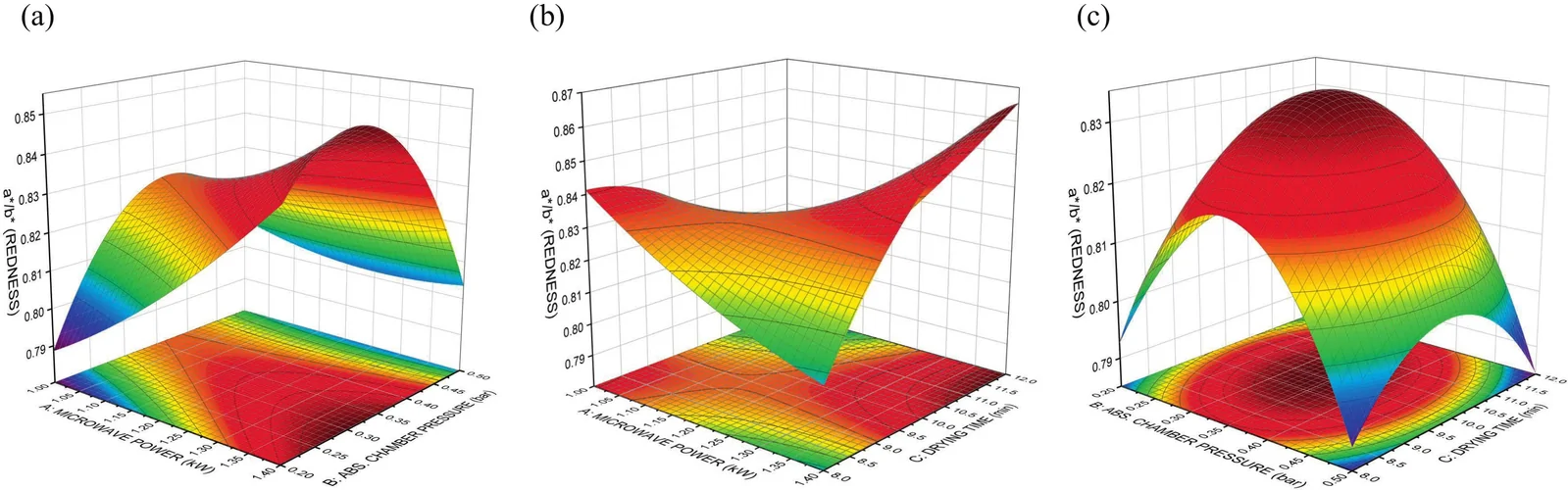
Response surface plots showing the effect of MWV drying parameters on color *a*^⁎^/*b*^⁎^ of tomato snack bar (a, b, and c).

The 3D plots in Fig. 4 show the relationship of the *a*^⁎^/*b*^⁎^ ratio on tomato snack bars, ranging from 0.766 to 0.876. Although the overall quadratic model for *a*^⁎^/*b*^⁎^ was statistically significant (*p* < 0.001), the significant lack of fit (*p* < 0.001) indicates that the model does not adequately describe the response over the entire experimental domain. Therefore, the response surface was not considered adequate for precise prediction across the complete experimental domain, and Fig. 4 is presented only as a qualitative visualization of the observed, condition-dependent color trends.

The results demonstrated that the effect of microwave power depended on drying time. The experimental data also showed combination-dependent behavior: at 0.35 bar and 12 min, *a*^⁎^/*b*^⁎^ was higher at 1.4 kW than at 1.0 kW, whereas at 8 min the direction was reversed. Therefore, microwave power did not have a uniform effect on the color ratio across the investigated conditions. Absolute chamber pressure also showed a nonlinear relationship with *a*^⁎^/*b*^⁎^ ratio in the fitted surface. However, the apparent curvature and location of the predicted maximum should be interpreted cautiously because of the significant lack of fit.

Changes in *a*^⁎^/*b*^⁎^ ratio during drying may be associated with several mechanisms occurring concurrently, including carotenoid degradation or isomerization, nonenzymatic browning, and changes in the physical structure and optical properties of the product (Liu et al., 2026). For instance, Heredia et al. (2010) reported that increased microwave power and temperature promoted lycopene degradation and isomerization in tomato and produced more orange and less red coloration. On the other hand, pigment composition, internal temperature, browning products, microstructure, and optical scattering were not measured in the present study. Therefore, the observed color trends cannot be attributed conclusively to lycopene degradation, Maillard reactions, hot-spot formation, puffing, or other individual mechanisms.

Because of the significant lack of fit in the *a*^⁎^/*b*^⁎^ model, color acceptability at the selected processing condition was assessed using the experimentally measured *a*^⁎^/*b*^⁎^ ratio rather than relying on global predictions from the quadratic model. Further measurements of carotenoids, browning markers, temperature distribution, and microstructure would be required to establish the mechanisms responsible for the observed color changes, as also stated by Song et al. (2017).

### 3.6 Effect of independent variables on lycopene content

**Fig. 5:**
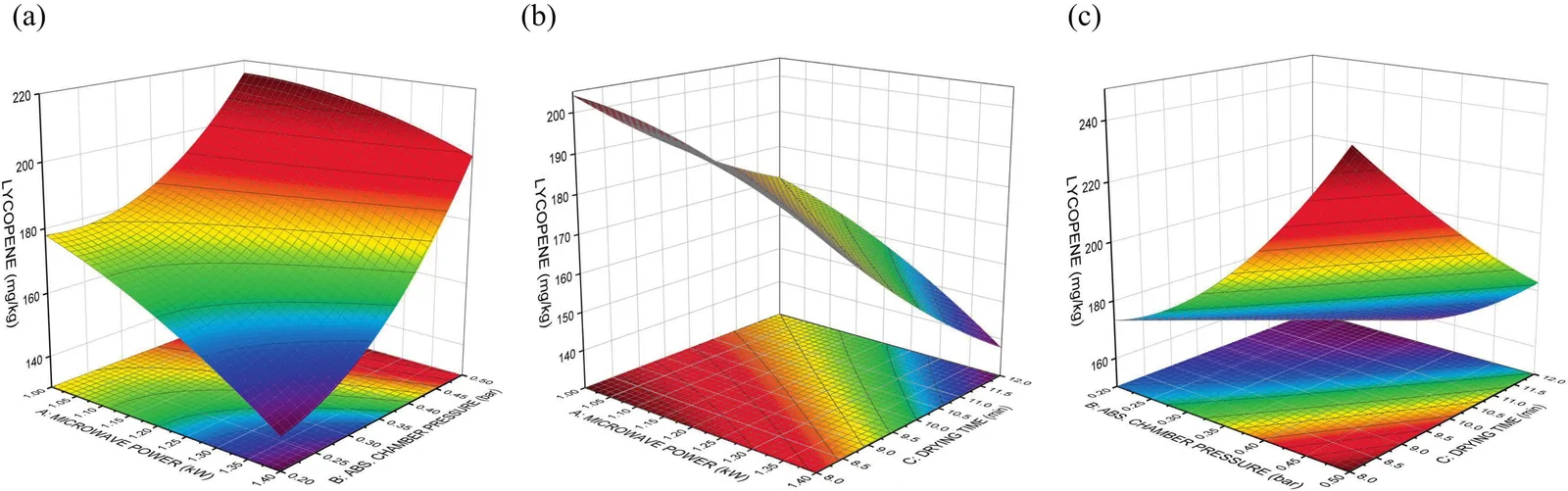
Response surface plots showing the effect of MWV drying parameters on lycopene content (mg/kg) of tomato snack bar (a, b, and c).

The effects of the independent variables on lycopene content in snack bars are shown in Fig. 5. The lycopene model was significant and had a nonsignificant lack of fit (*p* = 0.456), indicating that it adequately represented the experimental data within the investigated factor ranges. Absolute chamber pressure (B) had a nonlinear effect on lycopene content, as indicated by its quadratic term (+540) and the corresponding surfaces. Higher absolute chamber pressure, that is, applying a weaker vacuum, generally increased the extractable lycopene levels (Fig. 5(a) and 5(c)), which was also stated in another study (Gul et al., 2024). The highest lycopene content, 244.2 mg/kg dry matter, was obtained at 1.2 kW, 0.5 bar, and 8 min.

As lycopene was determined using solvent extraction, the observed increase represents a change in extractable lycopene content rather than directly demonstrated bioavailability. The higher boiling temperature at greater absolute chamber pressure may have permitted greater softening and disruption of the tomato matrix, thereby facilitating lycopene extraction, as previously suggested for heat-processed tomato matrices (Colle et al., 2010; Gul et al., 2024). Nevertheless, thermal processing can exert competing effects: matrix disruption may enhance lycopene extractability, whereas excessive thermal exposure can promote oxidation, isomerization, and degradation (Heredia et al., 2010; Mendelová et al., 2013).

The response surfaces indicated that the effects of microwave power and drying time depended on their combination with chamber pressure. At low absolute chamber pressure, increasing microwave power and extending the drying period generally reduced lycopene content. Although the stronger vacuum would initially promote evaporation at a lower temperature, rapid moisture depletion at high microwave power may reduce evaporative cooling during the later stages of drying and permit localized overheating. Microwave-vacuum drying has nevertheless been reported to produce high extractable lycopene contents under appropriately selected power and pressure combinations (Abano et al., 2013). Since the product temperature, oxygen concentration, matrix microstructure, and lycopene isomer distribution were not directly measured in the present study, the proposed mechanisms should be regarded as plausible explanations rather than experimentally confirmed pathways. Overall, the results demonstrate the importance of optimizing the selected parameters to balance efficient moisture removal with lycopene preservation.

### 3.7 Effect of independent variables on hardness value

**Fig. 6:**
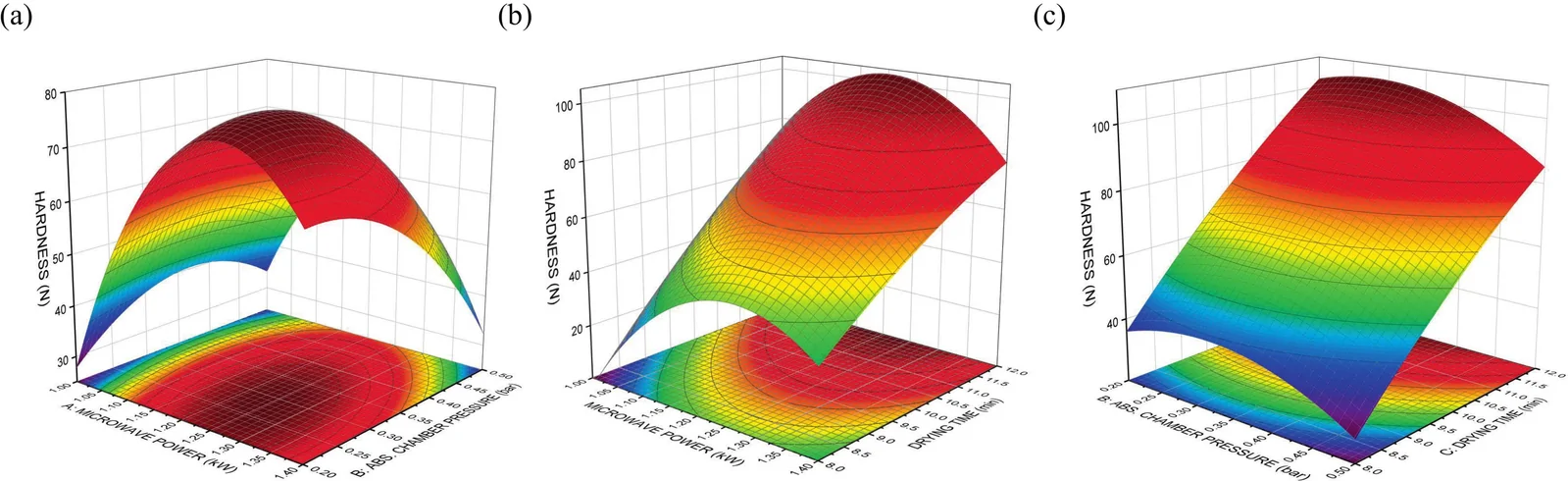
Response surface plots showing the effect of MWV drying parameters on the Hardness (N) of the tomato snack bar (a, b, and c).

The effect of variables on snack bar hardness is shown in Fig. 6. The experimentally measured hardness ranged from 14.71 to 113.24 N. Longer drying times (C) generally resulted in greater hardness and were accompanied by reductions in moisture content and *a*_w_. This relationship is consistent with reduced water plasticization and increased firmness as water is removed from a food matrix. However, as the glass-transition temperature and water-sorption behavior were not measured, a glass-transition-based mechanism cannot be confirmed for the present samples.

Although the quadratic hardness model was statistically significant, it exhibited significant lack of fit (*p* < 0.001). Thus, the model was not considered adequate for precise prediction, and the response surfaces in Fig. 6 are presented only as qualitative visualizations of the experimental trends. Within the fitted equation, the microwave-power terms and the interaction between microwave power and absolute chamber pressure indicated a nonlinear and condition-dependent relationship with hardness (Table 3). Nevertheless, the apparent maximum around 1.3 kW should not be interpreted as an accurately predicted optimum due to significant lack of fit.

The measured results suggest that the increase in hardness with drying time was primarily associated with moisture removal and the resulting reduction in water plasticization. Previous studies have shown that moisture loss, Maillard reactions, and protein aggregation may contribute to the hardening of high-protein bar systems (Zhou et al., 2013). Microwave heating may also cause uneven temperature and moisture distributions, while rapid vapor generation can affect the structure and texture of dried foods (Kumar & Karim, 2017; Zhang et al., 2006). However, these mechanisms cannot be established in the present study since glass-transition behavior, product temperature, Maillard reaction products, porosity, shrinkage, and microstructure were not measured. Consequently, the nonlinear effects of microwave power and absolute chamber pressure are interpreted as empirical, condition-dependent trends rather than evidence of caramelization-induced plasticization, puffing, or microcrack formation. Differential scanning calorimetry and water-sorption analysis would be required to evaluate glass-transition-related changes, while microscopy or porosity analysis and suitable chemical markers would be needed to confirm puffing, microcrack formation, and caramelization or Maillard-reaction-related effects (Zhang et al., 2006).

At the selected processing condition, the experimentally measured hardness was 54.35 N. This value was used to determine whether the product satisfied the predefined local texture criterion rather than to validate the overall quadratic hardness model.

### 3.8 Effect of independent variables on energy expenditure

**Fig. 7:**
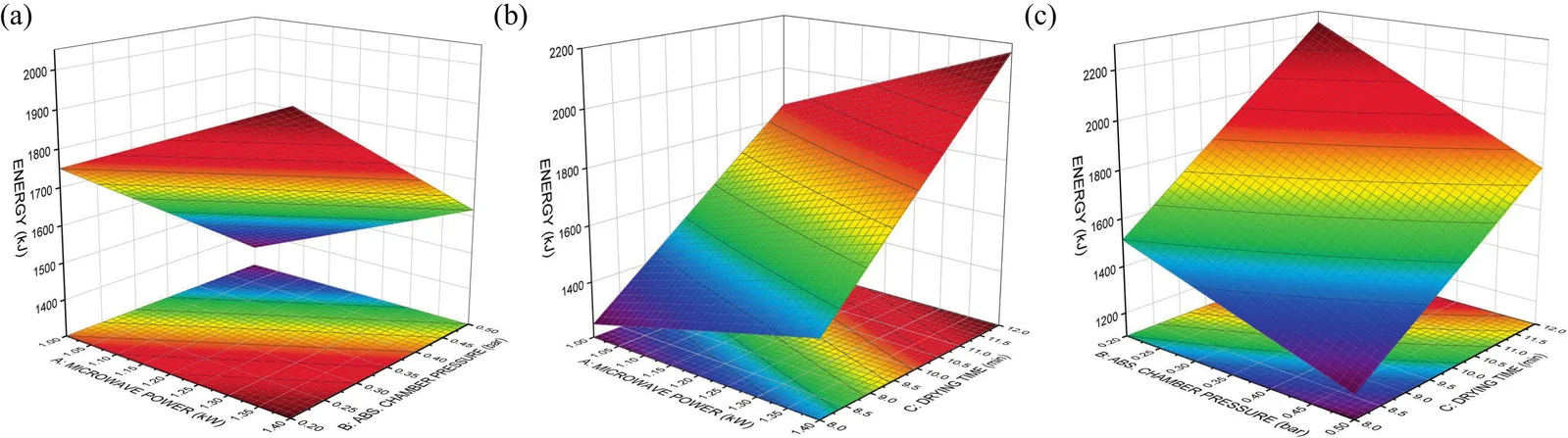
Calculated energy-consumption surface plots showing the effect of MWV drying parameters on the estimated energy consumption (kJ) of the tomato snack bar (a, b, and c).

The calculated effects of independent variables on energy during snack bar drying are shown in Fig. 7. This figure displays the deterministic energy objective calculated from the equipment-rated power model, not an empirically fitted RSM response. Thus, energy was used as an objective function in the numerical optimization. Accordingly, no ANOVA, coefficient of determination, or lack-of-fit statistics were calculated for energy.

Within the deterministic calculation, drying time had the greatest effect on estimated energy consumption. Longer drying increased energy notably, and microwave power's impact grew with extended drying (Fig. 7(b)). Absolute chamber pressure was inversely related to energy because vacuuming costs energy to lower the absolute pressure inside the microwave (Fig. 7(c)). Lower absolute pressure (stronger vacuum) requires more air removal to achieve faster drying, increasing estimated energy use. The interaction between absolute chamber pressure and drying time reflected the dependence of the vacuum-system energy requirement on the duration of operation.

The lowest energy among the runs was 1162 kJ with 1.2 kW microwave power, 0.50 bar absolute chamber pressure, and 8 min drying time. Since no direct power meter was used, these values should be interpreted as theoretical estimates for within-study optimization rather than measured energy efficiency. Direct electrical measurements of the microwave generator, vacuum pump, cooling system, standby consumption, and other system losses would be required before drawing conclusions regarding industrial energy efficiency, scale-up, or environmental performance.

### 3.9 Optimization

Table 4 compares the model predictions with the experimental validation at the selected compromise condition (1.28 kW, 0.46 bar absolute pressure, and 9.2 min). All variables were kept within their ranges, targeting water activity (0.5), moisture (15.0% db), and hardness (45 N). Lycopene and color *a*^⁎^/*b*^⁎^ were maximized.

**Table 4:** Predicted and experimental validation values at the selected compromise condition.

Condition	Factors			Measured responses					Calculated objective
	A (kW)	B (bar abs.)	C (min)	*a*_w_	Moisture (% db)	*a*^⁎^/*b*^⁎^	Lycopene (mg/kg dry)	Hardness (N)	Energy estimate (kJ)
Predicted	1.28	0.46	9.2	0.501	15.01	0.811	206.0	51.81	1424
Experimental	1.28	0.46	9.2	0.494	15.15	0.806	216.5	54.35	1424

*Note: Relative experimental deviations from the predicted values were −1.4% (a*_w_*), +0.9% (moisture), −0.6% (a*^⁎^/*b*^⁎^*), +5.1% (lycopene), and +4.9% (hardness). The factor settings shown are rounded; the reported energy estimate was calculated from the unrounded optimizer settings and is identical in both rows because it is deterministic, not experimentally fitted. Significant lack of fit for a*^⁎^/*b*^⁎^*and hardness limits validation of those responses to this selected condition*.

Experimental validation yielded a water activity of 0.494, moisture of 15.15%, an *a*^⁎^/*b*^⁎^ ratio of 0.806, lycopene content of 216.5 mg/kg dry matter, and hardness of 54.35 N. Relative deviations from the model predictions were − 1.4% for *a*_w_, +0.9% for MC, −0.6% for *a*^⁎^/*b*^⁎^, +5.1% for lycopene, and + 4.9% for hardness. These results indicate generally good agreement between the predicted and experimental responses at the selected condition. In particular, the agreement observed for *a*_w_, moisture content, and lycopene supports the predictive adequacy of their quadratic models near the selected condition.

The six-center runs conducted at 1.2 kW, 0.35 bar, and 10 min gave a mean *a*_w_ of 0.501 ± 0.012, essentially matching the optimization target of 0.500. The *a*^⁎^/*b*^⁎^ and hardness results demonstrated that the selected product satisfied the predefined local quality criteria only for selected optimum conditions. However, agreement at the selected condition does not eliminate the significant lack of fit observed for the corresponding quadratic models or establish their predictive reliability throughout the full experimental region. Accordingly, these two models were used only to identify qualitative trends and assess the product locally at the selected condition. Similarly, the overall desirability value of 0.83 represents a numerical compromise among the response objectives and should not be interpreted as evidence that every fitted response surface was statistically adequate. Although a*/b* and hardness were retained in the desirability optimization, their contribution was interpreted only as local empirical guidance rather than globally predictive optimization criteria.

The theoretical energy estimate was 1424 kJ at the selected condition. As energy was calculated deterministically from the equipment-rated power model rather than measured directly, it was used only as an optimization objective and was not subjected to RSM fitting or experimental model validation. Abano et al. (2012) reported that MVD affected both moisture removal and extractable lycopene content in tomato products. Microwave-vacuum processing has also been reported to achieve rapid moisture removal compared with conventional drying approaches (Abano et al., 2012, 2013; Gul et al., 2024). Although direct comparison among studies is limited by differences in product formulation, sample geometry, equipment, and drying endpoints, the selected condition in the present study reduced the snack bar moisture content to approximately 15% within 9.2 min. This result demonstrates the rapid processing potential of microwave-vacuum drying while maintaining the selected water activity, moisture, color, lycopene, and texture criteria.

## 4 Conclusion

This study optimized the microwave-vacuum drying of tomato snack bars using response surface methodology, with variables including microwave power, absolute chamber pressure, and drying time. The parameters were optimized using quadratic models based on the Box–Behnken design. It showed that all factors influenced dehydration, physicochemical, and textural properties, as well as energy consumption. The quadratic models for *a*_w_, moisture, and lycopene were adequate within the studied domain. By contrast, significant lack of fit limited the *a*^⁎^/*b*^⁎^ and hardness surfaces to exploratory trend interpretation, though their experimental values were acceptable at the selected condition. Energy was used only as a deterministic optimization objective and not as an empirical RSM response. Microwave-vacuum drying shortened processing time and maintained high lycopene content, highlighting its efficiency. Overall, the findings provide insights into the drying behavior and properties during microwave drying of tomato snack bars and other similar fruit- and vegetable-based products. Thus, they offer valuable guidance for future research on drying methods and industry practices to enhance quality and sustainability. However, validation through direct energy metering, temperature mapping, microstructural and thermal analyses, sensory testing, and storage studies is needed before scale-up or broader quality and sustainability claims.

## Declaration of Generative AI and AI-assisted technologies in the manuscript preparation process

During the preparation and revision of this work, the authors used ChatGPT (OpenAI) to assist with background literature searches, identification of potentially relevant references, and improvement of the language, clarity, and organization of selected manuscript passages and responses to reviewers. Grammarly was also used to assist with language editing and improve the wording of a limited number of passages. All suggested references were independently checked and verified against the original sources by the authors. After using these tools, the authors reviewed and edited the content as needed and take full responsibility for the content of the published article.

## CRediT authorship contribution statement

**Muhammed Rasim Gul:** Writing – review & editing, Writing – original draft, Visualization, Software, Methodology, Investigation, Formal analysis, Data curation, Conceptualization. **Nalan Yazicioglu:** Writing – review & editing, Software, Formal analysis. **Gulum Sumnu:** Writing – review & editing, Supervision, Conceptualization. **Mecit Halil Oztop:** Writing – review & editing, Supervision, Resources, Project administration, Funding acquisition, Conceptualization.

## Declaration of competing interest

The author(s) declare(s) that the study does not involve humans nor animal subjects.

## Data Availability

Data will be made available on request.
